# Composition, Diversity, and Biomass of Scarabaeoidea in Tropical Dry Forests of the Colombian Caribbean Region

**DOI:** 10.1007/s13744-026-01382-x

**Published:** 2026-04-15

**Authors:** Carlos Taboada-Verona, Rafael Narváez, Jorge Ari Noriega

**Affiliations:** 1 Universidad del Pacífico, Buenaventura, Colombia; 2 Universidad de Sucre, Sincelejo, Colombia; 3 Grupo de Agua, Salud y Ambiente, Facultad de Ingeniería, Universidad El Bosque, Bogotá, Colombia

**Keywords:** Beta diversity, Conservation, Dung beetles, Functional groups, Passalidae, Pitfall traps, Scarabaeidae

## Abstract

Knowing the spatial variability of a region helps us develop specific conservation strategies to mitigate human-induced disturbances. In this context, beetles are excellent bioindicators of spatial changes resulting from human disturbance. This work aims to determine the spatial heterogeneity of the diversity of the superfamily Scarabaeoidea in dry forests of the Colombian Caribbean region. Individuals were captured using baited flight interception traps, pitfall traps, fruit-baited traps, manual collection, and light traps. Forty-seven species, 23 genera, and four families were recorded; the dominant species were *Sylvicanthon aequinoctialis* (Harold, 1868), *Eurysternus caribaeus* (Herbst, 1789), and *Ateuchus* sp. 1. Sampling completeness was above 99%. The range-abundance curves show that the structure is similar between the three sampling sites, presenting very steep slopes, indicating low equity. The dominant trophic guilds were coprophages, constituting 53% of all species, followed by necrophages and sapro-xylophages, with 17% and 15%, respectively. Regarding alpha diversity, the sites showed a pattern of high richness, while beta diversity showed a dissimilarity of 38%. For biomass, no significant differences were detected. The variation in diversity in the study sites may be related to low spatial heterogeneity. Some families are subject to spatio-temporal variability, reproduction cycles, and trap effectiveness. Although this region is under different anthropogenic pressures, such as deforestation, livestock, tourism, and monocultures, the beta diversity of the superfamily Scarabaeoidea was homogeneous in terms of the pattern in the sites studied here. Finally, the composition, diversity, and biomass of the subfamily Scarabaeoidea are potential indicators of spatial heterogeneity in disturbed landscapes.

## Introduction

The Seasonally Dry Neotropical Forest (SDNF) presents a discontinuous distribution, mainly associated with climatic conditions with temperatures above 17 °C, an annual precipitation close to 1800 mm, and a pronounced dry season (i.e., 3 to 6 months) where the plants are mainly deciduous (DRYFLOR et al. 2016). Due to its adaptations to extreme climate variations, these conditions have made this ecosystem one of the most complex in the Neotropics (Linares-Palomino et al. 2011). In Colombia, the SDNF originally covered around 8,882,854 ha; however, 90% of its cover has been replaced by pastures, agricultural fields, and urbanization by the end of the twentieth century (Pizano et al. 2014). According to the above, it has been recognized as a highly threatened ecosystem (Pizano et al. 2014). Currently, only 720,000 ha of original cover remain, 332,810 ha represented in continuous forest areas, and 384,416 ha belong to successional mosaics (García et al. 2014). In Colombia, only 4% of the dry forest surface remains relatively intact, and of this area, only 5% is conserved in protected areas (García et al. 2014).

The most critical forest relics of this ecosystem are distributed in the Caribbean, Norandina region, inter-Andean valleys of the Magdalena, Cauca, Patía rivers, and in the eastern plains of the departments of Arauca, Casanare, Meta, and Vichada (IAVH 1998; Galvis et al. 2014). In this sense, the largest, best preserved, and most connected fragments are found in the Caribbean region, specifically in the Montes de María, which includes the departments of Sucre and Bolívar (Pizano et al. 2014). However, in the last two decades, the imminent advance of the agricultural, mining, and livestock frontier has decreased its coverage and diversity (Sampedro et al. 2014; Olascuaga-Vargas et al. 2016).

Another significant problem of this region is the few studies that allow us to know its diversity and, therefore, propose conservation strategies for the fauna and flora; some have only focused on vertebrates and vascular plants (Galván-Guevara et al. 2009; Sampedro et al. 2013; Herazo et al. 2017); but other groups are little known, such as insects, which are considered the most diverse taxa on earth (Tihelka et al. 2021). These include the superfamily Scarabaeoidea, represented by more than 41,000 species (Schoolmeesters 2024) and considered one of the most diverse groups within the order Coleoptera (Scholtz and Grebennikov 2005). Furthermore, their value as a potential bioindicator tool has been established (Halffter and Favila 1993; Schuster et al. 2000; Otavo et al. 2013).

Some studies in the Montes de María department of Sucre, Colombia, have focused on taxonomic lists, such as the work carried out by Navarro et al. (2011a) for the subfamily Scarabaeinae. Likewise, these same authors studied the seasonal variation of this subfamily in a dry forest fragment and an area of livestock use (Navarro et al. 2011b). For their part, Bohórquez and Montoya (2011) analyzed the population fluctuation and trophic preference of *Dichotomius belus* (Harold, 1880). Taboada-Verona et al. (2017) studied the distribution of *Ontherus brevipennis* Harold, 1867 in two localities of the Serranía Coraza reserve. Another family studied corresponds to Passalidae, where Taboada-Verona and Murillo-Ramos (2020) analyzed the richness and distribution in three subregions of the department of Sucre and included several localities in Montes de María. However, there are no local or regional studies that relate variables such as composition, diversity, and biomass to spatial heterogeneity, as is the case in recent studies in Latin America (Alvarado et al. 2018; Estupiñan-Mojica et al., 2022; Gomez-Cifuentes et al., 2025).

The Montes de María present climatic variations allowing the formation of different communities of woody plants whose floristic origin is associated with different biogeographic provinces, especially in Mesoamerica (Mercado-Goméz et al., 2019). In other words, the diversity of this area may become greater, so a deeper exploration is required to generate a better approximation of the diversity of the Scarabaeoidea in this region. However, it is essential to understand the degree of spatial heterogeneity to take action at the conservation level. Considering the above, we address the following research question: Can the composition, diversity, and biomass of the Scarabaeoidea superfamily be an adequate indicator of spatial heterogeneity in the dry forests of the Colombian Caribbean region? In this sense, we predict that the Scarabaeoidea superfamily will be a good bioindicator and that the values of richness and abundance will be similar, as there is high heterogeneity.

## Materials and methods

### Study area

The study was carried out in the department of Sucre, Colombian Caribbean. The area is framed in the tropical dry forest life zone, according to Holdridge (1947). Geomorphologically, this area belongs to the northern foothills of the San Jerónimo mountain range, reaching maximum heights of 800 m a.s.l. in Cerro Maco (department of Bolívar) and 600 m a.s.l. in the Pita hill (department of Sucre). This formation resulted from different tectonic events of the Miocene, giving rise to an area of hills and mountains known as Montes de María and Serranía de San Jacinto (Galván-Guevara et al. 2009). The Montes de María have a warm climate with an average temperature of 28 °C and a relative humidity that varies between 75% during dry periods and 85% during the rainy period. It has an annual rainfall of 1800 mm, with periods of rain from April to November, interrupted in July by a slightly dry period. Likewise, it has a prolonged period of drought between December and March with averages of 100 mm (Aguilera-Díaz 2013). Regarding the vegetation, the most important plant families are Fabaceae, Malvaceae, Meliaceae, Sapindaceae, Capparaceae, Rubiaceae, and Cactaceae, with the dominant species being *Ampelocera edentula* Kuhlm, *Aspidosperma polyneuron* Mull. Arg, *Brosimum alicastrum* Swartz, *Myrcia fallax* (Rich.) DC., and *Simira cordifolia* (Hook.F) Steyerm (Herazo et al. 2017).

### Field and lab phase

**Fig. 1:**
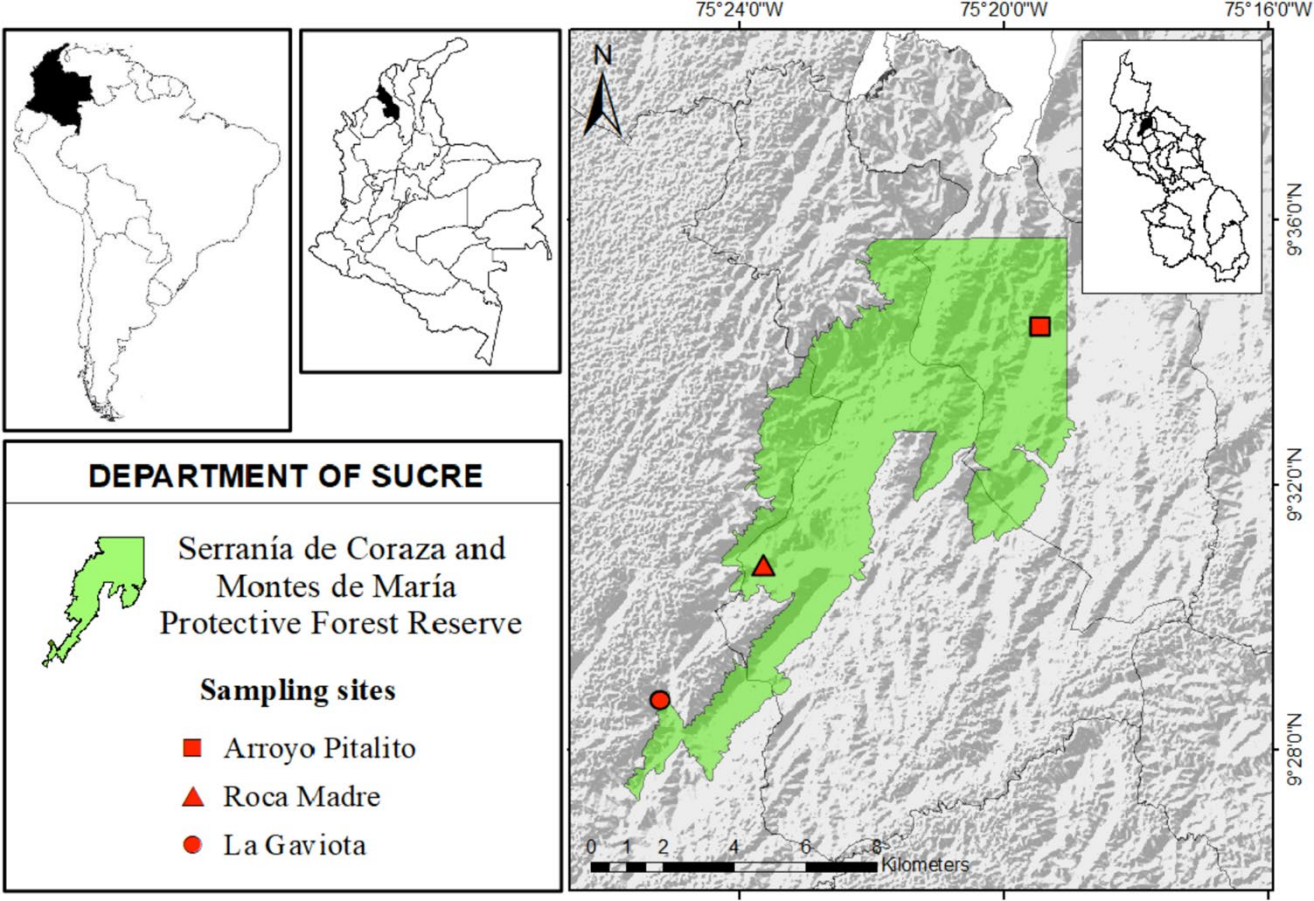
Location of the three sampling sites in the Neotropical dry forest in the Caribbean region of Colombia

The selection of the sampling areas was carried out, considering that each of the sites met the characteristics of the dry forests of the region, based on the descriptions of the methodology adopted for Colombia from the CORINE Land Cover methodology (Büttner and Kosztra 2017). This methodology includes choosing areas with extensive vegetation cover greater than 25 ha and non-anthropogenic vegetation with water availability throughout the year. According to the parameters mentioned above, taking into account the biotic and abiotic characteristics of each site (see Table 1), and through geographic information systems, three sites were analyzed and selected in the municipalities of Chalán (Arroyo pitalito, AP), Colosó (Roca Madre, RM), and Toluviejo (La Gaviota, LG), which represent a large part of the spatial heterogeneity of the region (Fig. 1).

**Table 1:** Characterization of the three different study sites (*AP* Arroyo Pitalito, *RM* Roca Madre, *LG* La Gaviota) according to biotic and abiotic variables of the Serranía Coraza Protective Forest Reserve, Colombian Caribbean

Characteristics (biotic and abiotic)	Study areas (sites)		
	Arroyo Pitalito (AP)	Roca Madre (RM)	La Gaviota (LG)
Municipalities	Chalan	Colosó	Toluviejo
Elevation	600–650	300–350	190–250
Forest type	Tropical Dry Forest	Tropical Dry Forest	Tropical Dry Forest
Canopy height	15–16	14–16	14–15
Predominant growth habit	Shrubby, with trees and vines	Shrubby, with trees and vines	Shrubby, with trees and vines
Main families	Rubiaceae, Moraceae, Meliaceae, Apocynacea, Fabaceae	Fabaceae, Moraceae, Apocynaceae, Rubiaceae, Euphorbiaceae	Moraceae, Urticaceae, Euphorbiaceae, Burseraceae
Annual mean temperature (°C)	24.7	26.3	27.3
Annual mean precipitation (mm)	1527	1485	1375
Humidity (%)	75–85	75–80	70–80
Soil type	Young sedimentary soils (Inceptisols and Entisols) dominated by sandy-clay structure, with an undulated and broken topography	Vertical sedimentary soils with a high sandy-clay and clay loam content, with an undulating and broken topography	Sedimentary soils with presence of limestone rocks, with vertical characteristics, dominated by clay-loam, and an undulating topography
Degree of disturbance	Medium–high	Medium-medium	Medium–high
Disturbance type	Anthropogenic (agricultural and tourist)	Anthropogenic (mining and livestock)	Anthropogenic (livestock and agricultural)
Activities or causes of disturbance	Monocultures, ecotourism, logging and burning	Mining (limestone extraction), ecotourism and livestock farming	Livestock farming, logging and burning
Ecological disturbance description	Alteration due to agricultural activities, burning and habitat fragmentation	Localized impact from mining, although there is some remaining vegetation cover	Alteration by grazing and deforestation with degraded open habitats

Data based on studies in the area (Olascuaga-Vargas et al. 2016; Herazo et al. 2017; Ballud et al. 2018; FAO and IIASA 2023)

Sampling was carried out from November to December 2017, sequentially at the three sites, maintaining similar climatic conditions. At each site, three linear transects of 1000 m were established at a distance of 500 m from each other, always located inside the forests, more than 500 m from the edge. Five different collection methods were implemented in each transect: (i) two flight interception traps (1.5 high × 6 m wide) baited with decaying fruit (500 g of semi-fermented banana) and decomposed fish (500 g) at the beginning and at the end of each transect; (ii) ten pitfall traps (200 ml) buried at ground level with 100 ml of soapy water and 25 g of bait (five baited with fresh human excrement and five with decomposing fish) in gauze suspended from a wire and spaced 50 m apart; (iii) ten fruit-baited traps of 4 L baited with 1 kg of decomposing fruit (i.e., a mix of banana, pineapple, papaya, and mango in equal proportions), suspended at a height of 2 m and separated 50 m from each other; (iv) manual capture collections were made in flowers, foliage, fungi, nests of social insects, and decomposing logs with a sampling effort of three man-hours in each transect; (v) one white light trap was set up from 6:00 p.m. to 10:00 p.m. for three consecutive days, at a distance of 200 m from the end of the transect (Larsen and Forsyth 2005; Luna 2005; Noriega and Fagua 2009; Solís, 2007; Mora-Aguilar et al., 2023). Sampling at each site was kept active for 3 days and was checked every 24 h.

Taxonomic identification was carried out at the species level using different taxonomic keys (Howden and Young 1981; Ocampo 2006; Smith and Skelley 2007; Edmonds and Zidek, 2010; Camero 2010; Jiménez-Ferbans and Amat-García, 2010; Vaz-de-Mello et al., 2011; Sanabria-García et al. 2012) and consultation with experts from each group. The collected individuals were deposited in the Zoological Museum of the University of Sucre (MZUSU) collection and in the reference collection of the last author (CJAN). Additionally, the biomass measurement was carried out by species and for the entire community in the three sampling sites, following the protocol proposed by several authors (Lobo 1993; Davis 1996; Noriega et al. 2021). Ten individuals of each species were randomly selected (five males and five females) and dehydrated in an oven (Binder FED 400) at 80 °C for 24 h. They were then weighed on a precision electronic scale (Precisa Series 165 BJ 1000 C), calculating each species’ average weight per individual.

### Data analysis

A rarefaction and extrapolation-interpolation curve were performed to calculate the expected number of species. This method uses the sample and a completeness curve prepared with twice the size of the smallest reference sample to be compared, with a 95% confidence interval, obtaining a resampling of 100 bootstrap pseudoreplicates (Chao et al. 2014). These analyses were implemented in the R package iNEXT (Hsieh et al. 2016) following the parameters established by Chao et al. (2014) and Colwell et al. (2012). To establish community dominance, range-abundance curves with log (10) transformation were made to compare each site’s species composition, abundance, and uniformity (Magurran 1989). To compare the sampling sites in terms of biomass, a Kruskal–Wallis test, a normality Shapiro–Wilk test, and a Bartlett test were performed to evaluate the homogeneity of variances.

To assess the representativeness of the sampling by location, considering that Scarabaeoidea families differ in their richness, abundance, collection method, and sampling effort, the sampling coverage method proposed by Chao and Jost (2012) was selected. This method allows for standardized comparisons between communities with different sampling efforts and levels of completeness, regardless of the type of trap used. This approach is especially useful when some families have limitations in abundance and richness, as it adjusts comparisons to the degree of effective coverage rather than nominal effort. In this way, the comparison between locations is made under an equivalent level of completeness, ensuring a more robust and objective assessment of total diversity.

To establish diversity, the true diversity indices (“Hill” numbers) expressed in the effective number of species (0D, 1D, 2D) were used (Jost 2006), which were calculated and graphed (profile of diversity) through the R package Entropart (Marcon and Hérault 2015). Jost (2006) proposes to analyze diversity at several levels that include zero-order diversity (0D) or species richness, first-order diversity (1D) or exponential of the Shannon index, which weights diversity by the relative abundance of species and diversity of order two (2D), or inverse of the Simpson index (Chao et al. 2014) that takes into account the most abundant species. To calculate how many times a set is more or less diverse than another in terms of its diversity estimates (qD ± 95% CI), the magnitude of the difference (MD) was calculated, which works under the same sample coverage and respect the replication principle. This measurement is calculated in percentage terms using the following equation: %MD = 100 − [(qD sample2 × 100)/qD sample1]. When sample 1 is more diverse than sample 2, the percentage of MD will be positive; otherwise, it will be negative. The MD varies between 0 (no change in pairwise diversity) and 100 (completely different diversity).

Beta diversity was calculated using the Sorensen index proposed by Baselga (2010). This index decomposes beta diversity into two different components, such as nestedness (βNES) and turnover (βSIM), which were calculated between sampling sites. This analysis was performed through the beta part package in R (Baselga and Orme 2012).

## Results

### Taxonomic composition

**Fig. 2:**
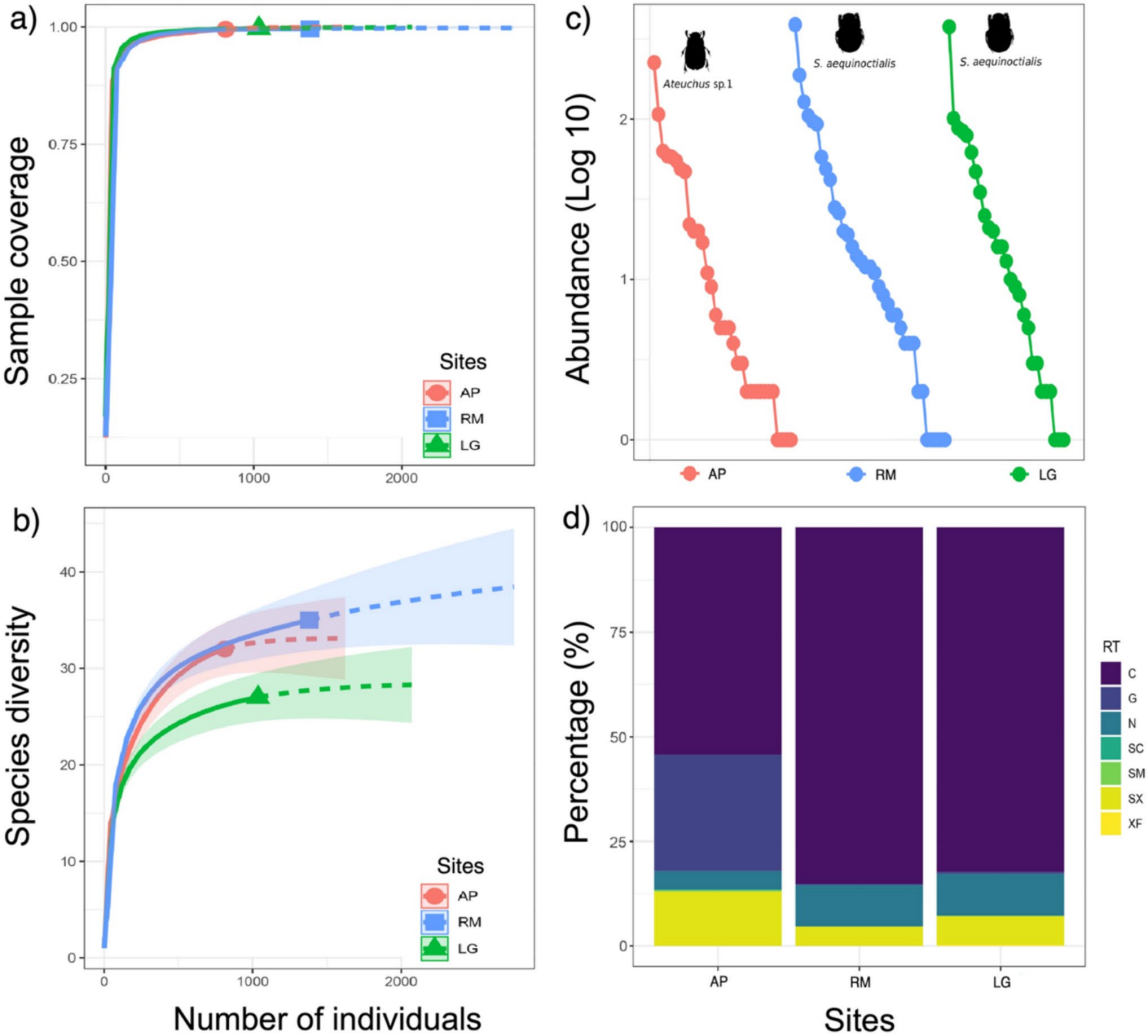
Rarefaction and interpolation–extrapolation curves, based on the superfamily beetles (Scarabaeoidea) in the three study sites; solid lines represent the estimation using interpolation, and dashed lines represent extrapolation. The shaded areas for each graph are the 95% confidence intervals: **a** sampling completeness, **b** richness estimation, **c** range-abundance curves, and **d** percentage of trophic guilds for each of the study sites (AP: Arroyo Pitalito, RM: Roca Madre, and LG: La Gaviota)

A total of 3228 individuals, 811 in Arroyo Pitalito, 1381 in Roca Madre, and 1036 in La Gaviota, were captured (Fig. 1). Forty-seven species, 23 genera, and four families of the superfamily Scarabaeoidea were recorded (Table 2). The dominant species for the three sites were *Sylvicanthon aequinoctialis* (Harold, 1868) (*n* = 871; 26.98%), *Eurysternus caribaeus* (Herbst, 1789) *(n* = 247; 7.65%), and *Ateuchus* sp. 1 (*n* = 233; 7.21%). The families with the highest abundance and richness values were Scarabaeidae (*n* = 34 spp.; 88.4%) and Passalidae (*n* = 7 spp.; 7.67%), while Hybosoridae (*n* = 1 spp.; 3.46%) and Melolonthidae (*n* = 5 spp.; 0.24%) present the lowest values. The Scarabaeinae consists of 14 genera. The genera *Canthon* and *Eurysternus* of the family Scarabaeidae had the greatest species richness (7 and 5 spp., respectively), while in the family Passalidae, the genus *Passalus* had the greatest richness (4 spp.). The completeness of the sample with a 95% confidence interval for the sampling sites was 99.5% for Arroyo Pitalito, 99.7% for La Gaviota, and 99.6% for Roca Madre. These values suggest that sampling was representative of all three sites (Fig. 2a). When extrapolating the number of individuals, significant differences in richness were recorded between sites (Fig. 2b).

**Table 2:** Abundance and richness of Scarabaeoidea species in three sites (*AP* Arroyo Pitalito, *RM* Roca Madre, *LG* La Gaviota) of the Serranía Coraza Protective Forest Reserve, Colombian Caribbean

Families	Species	RT	Sites			Total (%)
			AP	RM	LG	
Hybosoridae	*Anaides fossulatus* Westwood, 1846	N	2	97	13	112 (3.46)
Melolonthidae	*Cyclocephala brevis* Höhne, 1923	SM	2	0	0	2 (0.06)
	*Cyclocephala discolor* (Herbst, 1790)	SM	0	2	0	2 (0.06)
	*Leucothyreus* sp.	XF	0	1	0	1 (0.03)
	*Macraspis lucida* (Linnaeus, 1767)	XF	0	2	0	2 (0.06)
	*Strategus aloeus* (Linnaeus, 1758)	SC	1	0	0	1 (0.03)
Passalidae	*Passalus interruptus* (Linnaeus, 1758)	SX	0	0	25	25 (0.77)
	*Passalus interstitialis* Eschscholtz, 1829	SX	0	9	1	10 (0.30)
	*Passalus punctatostriatus* Percheron, 1835	SX	55	12	0	67 (2.07)
	*Passalus punctiger* Lepeletier and Serville, 1825	SX	49	13	6	68 (2.10)
	*Paxillus leachi* (MacLeay, 1819)	SX	0	49	0	49 (1.51)
	*Popilius marginatus* (Percheron, 1835)	SX	0	12	16	28 (0.86)
	*Veturius aspina* Kuwert, 1898	SX	2	0	0	2 (0.06)
Scarabaeidae	*Ataenius* sp.	C	0	1	0	1 (0.03)
	*Ateuchus* sp. 1	G	225	6	2	233 (7.21)
	*Ateuchus* sp. 2	G	1	0	1	2 (0.06)
	*Canthidium* cf. *angusticeps*	N	6	7	5	18 (0.55)
	*Canthidium* cf. *euchalceum*	C	4	5	2	11 (0.34)
	*Canthon cyanellus* LeConte, 1859	N	2	0	79	81 (2.50)
	*Canthon juvencus* (Harold, 1868)	C	2	20	84	106 (3.28)
	*Canthon septemmaculatus* (Latreille, 1812)	C	2	0	0	2 (0.06)
	*Canthon subhyalinus* Harold, 1867	C	5	26	20	51 (1.57)
	*Canthon* sp. 1	C	2	58	62	122 (3.77)
	*Canthon* sp. 2	N	1	16	3	20 (0.61)
	*Canthon* sp. 3	C	0	1	35	36 (1.11)
	*Coprophanaeus corythus* (Harold, 1863)	N	20	19	0	39 (1.20)
	*Coprophanaeus gamezi* Arnaud, 2002	N	0	0	1	1 (0.03)
	*Deltochilum eurymedon* Genier, 2012	C	0	1	0	1 (0.03)
	*Deltochilum guildingii* (Westwood, 1835)	C	20	14	21	55 (1.70)
	*Diabroctis cadmus* (Harold, 1868)	N	0	0	2	2 (0.06)
	*Eurysternus caribaeus* (Herbst, 1789)	C	59	188	0	247 (7.65)
	*Eurysternus foedus* Guérin-Méneville, 1844	C	9	6	0	15 (0.46)
	*Eurysternus impressicollis* Castelnau, 1840	C	1	4	10	15 (0.46)
	*Eurysternus marmoreus* Castelnau, 1840	C	0	1	0	1 (0.03)
	*Eurysternus mexicanus* Harold, 1869	C	0	28	0	28 (0.86)
	*Ontherus brevipennis* Harold, 1867	C	58	0	0	58 (1.79)
	*Onthophagus acuminatus* Harold, 1880	C	47	42	8	97 (3.00)
	*Onthophagus* cf.* rhinolophus*	N	5	0	0	5 (0.15)
	*Onthophagus lebasi* Boucomont, 1932	C	11	11	47	69 (2.13)
	*Onthophagus marginicollis* Harold, 1880	C	3	4	16	23 (0.71)
	*Phanaeus hermes* Harold, 1868	C	3	4	3	10 (0.30)
	*Phanaeus pyrois* Bates, 1887	C	22	0	0	22 (0.68)
	*Sylvicanthon aequinoctialis* (Harold, 1868)	C	107	388	376	871 (26.98)
	*Trichillidium* sp.	C	5	93	9	107 (3.31)
	*Uroxys* cf. *deavilai*	C	0	8	101	109 (3.37)
	*Uroxys* cf. *microcularis*	C	63	128	0	191 (5.91)
	*Uroxys* cf. *micros*	C	17	105	88	210 (6.50)
Abundance (No. Ind.)			811	1381	1036	3228
Richness (No. Spp.)			32	35	27	47

*RT* trophic role, *C* coprophage, *G* generalist, *N* necrophage, *SX* sapro-xylophagous, *SM* sapro-meliphagous, *XF* xylo-phytophages, *SC* sapro-caulophagous

### Analysis of community structure

**Fig. 3:**
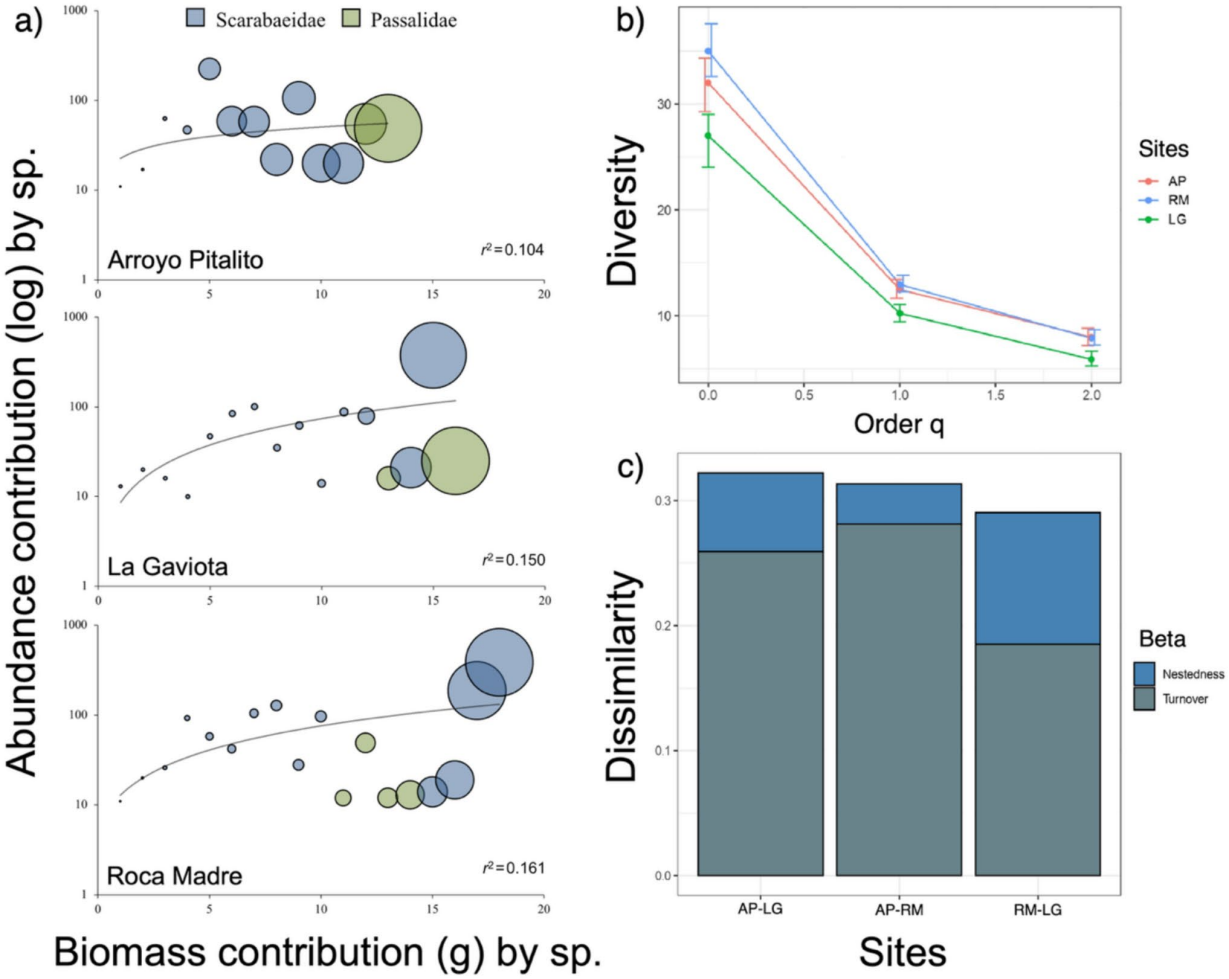
**a** Abundance contribution (log10) and biomass contribution for each family in the three sites, **b** alpha diversity profile for the three study sites, **c** variation of beta diversity, with percentage of turnover and nestedness, according to Jaccard dissimilarity index (AP: Arroyo Pitalito, LG: La Gaviota, and RM: Roca Madre)

The range-abundance analysis (Fig. 2c) shows that the structure is similar between the three sampling sites, presenting very pronounced slopes, which indicates low equity of species in all the sampled sites, although La Gaviota presented the lowest level of equity. The dominant trophic guild at all three sites was the coprophages, constituting 53% of all species, followed by the necrophages and sapro-xylophages with 17% and 15%, respectively, and the least abundant guild was sapro-caulophagous with 2% of the species and present only in Arroyo Pitalito (Fig. 2d). Regarding the total contribution to biomass, Arroyo Pitalito was the site with the highest contribution (78.91 g, 38.12%), with Scarabaeidae being the family that presents the highest contribution (43.59 g, 21.05%) followed by Passalidae (35.32 g, 17.06%). Roca Madre (69.08 g, 33.37%) is the second site where Scarabaeidae contributes 58.74 g (28.37%) and Passsalidae 10.34 g (4.99%). La Gaviota was the site with the lowest contribution (59.01 g, 28.50%), maintaining the same trend where Scarabaeidae contributed a greater value (33.70 g, 16.28%) and Passalidae the second place (25.30 g, 12.22%) (Fig. 3a). When comparing the three sites, no significant differences were detected (*H* = 3.329; *p* = 0.189; *α* = 0.05).

### Alpha and beta diversity

Roca Madre is the site with the greatest richness (35 spp.), followed by Arroyo Pitalito (32 spp.), and La Gaviota (27 spp.). This pattern is also seen in 1D, where Roca Madre and Arroyo Pitalito have 13 and 12 species, respectively, and La Gaviota only contains 10 species. For 2D, Arroyo Pitalito is the site with the highest number of dominant species (8 spp.), as Roca Madre (8 spp.), while La Gaviota presents the lowest value (6 spp.) (Fig. 3b). Given that the sampling coverage for the three sites was high (> 99%), it is possible to compare the magnitude of the difference (MD). For the analysis of beta diversity, the study determined that Arroyo Pitalito, compared to Roca Madre and La Gaviota, shared a dissimilarity value of 32%, while the latter two shared a 29%. The Sorensen dissimilarity index showed a total dissimilarity between the three sites of 38%, 30% explained by turnover between species and 7% by nesting phenomena (Bsor = 0.38, Bsim = 0.30, Bnes = 0.07) (Fig. 3c).

## Discussion

The great richness and abundance of the Scarabaeidae family found in this study can be attributed to the fact that it corresponds to one of the most diverse groups within the order Coleoptera (Nichols et al. 2007). This study reports a more significant number of species than those reported by Navarro et al. (2011a) for the Montes de María, 38% from the Colombian Caribbean (Noriega et al. 2013), and 12% from Colombia (Noriega et al. 2015). The high abundance of this group in the study sites may be the product of different factors, among which we can include (i) the reproductive cycles in the year because the majority of Scarabaeidae species are multivoltine, presenting more than two reproduction cycles per year (Morón et al. 1985) and (ii) the design of the traps allows high capture and collection efficiency (Ferrer-Paris et al. 2013), compared to the other families that have more univoltine habits and where the low efficiency of the traps for their capture has been demonstrated (Morón et al. 1985; Méndez-Aguilar et al. 2005). The genus *Canthon* presented the greatest richness; this may be because its species are considered very abundant and typical of dry forests in the Colombian Caribbean, with generalist habits that allow the use of the few existing resources (Jiménez-Ferbans et al. 2008; Martínez et al. 2009, 2010; Solís et al. 2011; Noriega et al. 2016).

In contrast, the Passalidae family presented 100% of the species reported for this region by Taboada-Verona et al. (2019) and Taboada-Verona and Murillo-Ramos (2020), 39% of the species reported for the Colombian Caribbean (Jiménez-Ferbans and Amat-García, 2009) and only 6% of those reported for Colombia (Jiménez-Ferbans et al. 2018). These results support what Schuster (1978) and Pardo-Locarno et al. (2000) stated when considering that dry tropical ecosystems have low richness in this group. The species with the highest abundance and collection frequency was *Passalus punctiger* Le Peletier and Serville, 1828. Pardo-Locarno et al. (2000) suggest that its wide distribution and high adaptability to temperature and humidity conditions make this species an abundant taxon in different ecosystems.

Regarding the Melolonthidae family, this represented 11.5% of the species recorded for this region and 3.92% of those present in the Colombian Caribbean (e.g., Restrepo-Giraldo et al. 2003; Pardo-Locarno et al. 2012; Orozco 2012; García-Atencia et al. 2015; López-García et al. 2015; Taboada-Verona et al. 2019). This family’s low richness and abundance can be explained by the fact that the emergence of adults is related to environmental variables, especially precipitation. Likewise, sampling throughout the year could show changes in the abundance and appearance of other groups of phytophages, such as Cetonines (Medida et al., 2018).

Concerning the range-abundance curves, the three sampling sites showed a dominant species; in the case of Arroyo Pitalito, it was *Ateuchus* sp. 1, which is a generalist genus with a broad ecological spectrum feeding on different types of dung, as well as carrion, fungi, and decomposing plants; some species have been recorded associated with cutter ant detritus and vertebrate burrows (Navarrete-Heredia 2001; Noriega and Calle 2008; Génier, 2015; Moctezuma et al. 2018), while in Roca Madre and La Gaviota, *S. aequinoctialis* was the species with the highest number of individuals collected, which is classified as a eurytopic species since it lives in a wide latitudinal and altitudinal range, being very abundant compared to the other species and presenting a wide distribution, being resistant to seasonal changes and reproducing throughout the year (Cupello and Vaz-de-Mello 2018). The lack of marked differences at the level of richness and abundance could be conditioning the result found with biomass, as evidenced in many studies that have demonstrated the marked correlation that exists between these variables (Braga et al. 2013; Griffiths et al. 2016; Correa et al. 2019; Milotic et al., 2019; Alvarado et al. 2020; Noriega et al. 2021). The ecological functions performed by the two main families of excrement removal, seed dispersal, and parasite control (Scarabaeidae; Nichols et al. 2008) and the degradation and recycling of wood as organic matter (Passalidae; Castillo and Reyes-Castillo 2003) seem to be unaffected by landscape heterogeneity.

Regarding alpha diversity, the site with the greatest richness was Roca Madre, followed by Arroyo Pitalito, which shares equal abundance and species dominance. High humidity in these sites allows the foliage to remain longer during periods of drought than in other forest fragments, producing a more significant amount of resources and a more constant presence of vertebrates throughout the year (Galván-Guevara and De la Ossa 2009; Galván-Guevara 2010; Sampedro et al. 2013), which generates a more stable supply of excrement. Likewise, this reserve is made up of a continuous forest cover (Galván-Guevara et al. 2009), which enables the constant presence of decomposing logs and thus facilitates the development of xylophagous and saproxylophagous families such as Passalidae and Melolonthidae. The site with the least richness was La Gaviota; despite being within the protected area, it exhibits the highest intensity of disturbance due to slash and burn processes for corn crops, presenting a shorter recovery time (Olascuaga-Vargas et al. 2016).

The analysis of beta diversity shows a low dissimilarity between the three locations, agreeing with the results obtained by Mercado-Goméz et al. (2021) for the Montes de María, where through beta diversity and multivariate analysis, they established three groups of woody plants. A group of these corresponds to the sites studied here; the low dissimilarity between them could be related to the slight climatic variation and their reduced geographical distance from each other (Mercado-Goméz et al., 2021), with an ecological similarity between the sites of sampling due to the continuity of its forests, which house 3000 ha of primary forest (Galván-Guevara et al. 2009; García-Martínez and Mercado-Goméz, 2017). The environmental filter has played an essential role in the formation of numerous communities (Emerson and Gillespie 2008), including beetles (Cottenie 2005; Betz et al. 2020), because it can limit the movement of species, even when there is reduced geographical distance and significant variation in abiotic characteristics between sites (Qian et al. 2005).

Many taxonomic groups are influenced by spatial heterogeneity due to sensitivity to alterations and modifications in the landscape, leading to changes in food preferences and dispersal capacity (Krebs 1972; García and Pardo 2004; Fahrig et al. 2011). The Serranía of Coraza forest reserve constitutes a landscape represented mainly by hills, with variations in temperature and precipitation, subject to constant pressure as a result of deforestation due to the establishment of areas for agriculture, pastures as well as hunting and extraction of wood with commercial value (Aguilera-Diaz, 2013). This has led in recent years to a reduction in vegetation cover, especially its primary forests, increasing the intervened areas by a large percentage (Ballud et al. 2018). These forests are found in a vegetation mosaic of forests, crops, and grazing-livestock areas (Olascuaga-Vargas et al. 2016). Despite this, there is a continuity of primary forest (Galván-Guevara et al. 2009), allowing species flow and the homogeneously maintained diversity. This suggests that anthropic perturbations (deforestation, livestock, monocultures, etc.) that generate habitat loss would not drastically affect the diversity and functionality but could influence other ecological changes, such as food preferences (Quintero and Roslin 2005; Fahrig et al. 2011).

Finally, even though the diversity and biomass of the superfamily Scarabaeoidea were homogeneous in the study area, some families are subject to variation in the availability of resources, reproduction cycles, and effectiveness of traps. In future studies, exploring how suitable proxy biomass is for diversity is necessary, especially when analyzing different trophic and taxonomic groups. Although this study shows that Scarabaeoidea is a good indicator of heterogeneity, comparisons with other taxonomic groups would be necessary. Even though spatial heterogeneity is not negatively affected by the various types and levels of anthropogenic disturbance in the region, which may still be very low, it is necessary to create conservation strategies and prioritize the maintenance of the connectivity of the mountain system and the forest cover. Low levels of spatial heterogeneity would allow the generation of generalized and scalable conservation strategies for the entire region, reducing management costs. Likewise, it is crucial to work with communities and promote more biodiversity-friendly production systems, such as agroforestry practices, which would help maintain Scarabaeoidea populations that are subject to habitat fragmentation. In addition, more sampling locations must be intensified throughout time to determine the dynamics of this diversity pattern. Knowing how this superfamily is represented will allow guidelines to be established for its conservation.

## Data Availability

The data that support the findings of this study are available from the corresponding author upon reasonable request.
